# Antiparasitic Action of *Lactobacillus casei* ATCC 393 and *Lactobacillus paracasei* CNCM Strains in CD-1 Mice Experimentally Infected with *Trichinella britovi*

**DOI:** 10.3390/pathogens11030296

**Published:** 2022-02-25

**Authors:** Zsolt Boros, Mihai Horia Băieș, Dan Cristian Vodnar, Călin Mircea Gherman, Silvia-Diana Borșan, Anamaria Cozma-Petruț, Menelaos Lefkaditis, Adriana Györke, Vasile Cozma

**Affiliations:** 1 Department of Parasitology and Parasitic Diseases, Faculty of Veterinary Medicine, University of Agricultural Sciences and Veterinary Medicine, 400372 Cluj-Napoca, Romania; zsolt.boros@usamvcluj.ro (Z.B.); mihai-horia.baies@usamvcluj.ro (M.H.B.); calin.gherman@usamvcluj.ro (C.M.G.); silvia.borsan@usamvcluj.ro (S.-D.B.); adriana.gyorke@usamvcluj.ro (A.G.); vasile.cozma@usamvcluj.ro (V.C.); 2 Department of Food Science, Faculty of Food Science and Technology, University of Agricultural Sciences and Veterinary Medicine, 400372 Cluj-Napoca, Romania; dan.vodnar@usamvcluj.ro; 3 Institute of Life Sciences, University of Agricultural Sciences and Veterinary Medicine, 400372 Cluj-Napoca, Romania; 4 Department of Bromatology, Hygiene, Nutrition, Faculty of Pharmacy, “Iuliu Haţieganu” University of Medicine and Pharmacy, 400349 Cluj-Napoca, Romania; 5 Laboratory of Microbiology and Parasitology, Department of Veterinary Medicine, School of Health Sciences, University of Thessaly, 43100 Karditsa, Greece; mlefkaditis@vet.uth.gr; 6 Academy of Agricultural and Forestry Sciences Gheorghe Ionescu-Siseşti (A.S.A.S.), 011464 Bucharest, Romania

**Keywords:** mice, probiotics, *Trichinella britovi*

## Abstract

Nematodes of the genus *Trichinella* are among the most widespread parasites of domestic and wild omnivores and predatory animals. The present study aimed to evaluate the antiparasitic effect of *Lactobacillus casei* ATCC 393 (original) and *L. paracasei* CNCM in CD-1 mice experimentally infected with *Trichinella britovi*. Four groups of 20 mice (10 females and 10 males/group) were used, with two control (C) groups and two experimental (E) groups, in which each animal received a daily oral dose of 100 µL of 10^5^ CFU/mL probiotics in Ringer’s solution. On day 7, all mice (except the negative control group) were infected orally with *Trichinella* (100 larvae/animal) as well as the two probiotics. On day 9 post-infection (p.i.), 10 mice/group were euthanized, and the presence of adult parasites in the intestinal content and wall was tested. On day 32 p.i., 10 mice/group were euthanized, then trichinoscopy and artificial digestion were performed to assess the muscle infection with *T. britovi*. On day 9 p.i., the experimental group pretreated with *L. casei* ATCC 393 (6.3 ± 3.03) showed a significantly lower number of adult parasites in the intestinal wall compared with the positive control group (24.6 ± 4.78). Additionally, a significantly lower adult parasite count in the intestinal wall was registered in female mice pretreated with *L. paracasei* CNCM (7.4 ± 4.71) compared to female mice from the positive control (29.0 ± 5.17). No statistically relevant results were obtained concerning the male mice or the data obtained at 32 days p.i., irrespective of mice gender.

## 1. Introduction

Trichinellosis is a zoonotic disease caused by nematodes of the genus *Trichinella*, which are among the most widespread parasites of domestic and wild omnivores and predatory animals [1]. Hosts can acquire infection by the consumption of raw or undercooked infected meat. The adults and larvae of *Trichinella* spp. reside in the same host, causing various adverse effects [2]. To date, two species of *Trichinella* have been reported in Romania—namely, *T. spiralis* (in domestic mammals) and *T. britovi* (in wild animals), but co-infections can also occur [3]. Trichinellosis is still present in Romania due to the local traditions and culinary customs that favor the transmission to humans. Humans can become infected through the consumption of untested raw meat, especially pork and wild boar [4,5], which has led to multiple outbreaks over the years [6,7]. The treatment of trichinellosis includes the use of benzimidazole derivates, such as albendazole or mebendazole [8,9]. Recently, the interest in developing different anthelmintic treatments that are both efficacious and safe has been increasing. Particular attention has been given to the potential anti-parasitic effect of probiotics [10].

Probiotics are live microorganisms that can provide a health benefit to the host when administered in adequate amounts [11]. Among them, lactobacilli are the most commonly used probiotics [12]. The *Lactobacillus* genus contains over 180 Gram-positive, anaerobic or microaerophilic, rod-shaped, non-spore-forming bacteria species [13]. 

Several studies suggest that probiotics can decrease the pathogenicity of parasites and, consequently, influence the course of parasitic infections [14]. In this regard, probiotics’ main mechanisms of action are related to their ability to compete with pathogens for adhesion sites, enhance intestinal mucosal barrier activity, produce antimicrobial agents, and regulate the host’s immune responses [15,16]. A good example, in this case, is an in vivo study conducted on gerbils (*Meriones unguiculatus*) that were experimentally infected with *Gardia lamblia* trophozoites and treated with 10^8^ CFU of *Lactobacillus johnsonii* La1 for seven days prior to parasite inoculation. During the study, no morphological damage to the intestinal epithelium was observed, but a reduction in active trophozoites and infection length was noticed [17]. Regarding nematodes, oral supplementation with *Lactobacillus rhamnosus* (JB-1) at a dose of 1 × 10^9^ CFU/day significantly accelerated *Trichuris muris* larvae removal in mice. This was accompanied by the upregulation of anti-inflammatory cytokine IL-10 levels and mucus-secreting epithelial cell numbers [18]. The positive effects of probiotic bacteria, such as reducing the parasite burden and pathological changes in experimental trichinellosis, have been previously described in several studies [19,20,21,22,23].

The most efficient manner of keeping the human population safe against trichinellosis is to eliminate the positive animals from human consumption. The use of probiotics in this parasitic infection is still in its infancy and they can only be used in experimental models. However, little is known concerning the effect of probiotics on species of *Trichinella* other than *T. spiralis*. Therefore, the present study aimed to evaluate the antiparasitic effect of two different probiotic strains of lactobacilli, *Lactobacillus casei* ATCC 393 (original) and *L. paracasei* CNCM (commercial product), in CD-1 mice experimentally infected with *T. britovi*. 

## 2. Results

The mean number of adults (9 days p.i.) and larvae (32 days p.i.) of *T. britovi* in the experimental groups can be seen in Table 1 (for all mice), Table 2 (for female mice), and Table 3 (for male mice). On the 9th and 32nd days after *T. britovi* infection (p.i.), the number of adult parasites and larvae found in the intestines (content and walls) and muscle (by trichinoscopy and artificial digestion) of CD-1 mice was higher in the positive control group than in the experimental groups that were pretreated with one of the two probiotic strains (Table 1, Table 2, Table 3). However, significant differences were registered for the number of adult parasites in the intestinal wall only in mice pretreated with *L. casei* (per total, and in female mice) and in female mice pretreated with *L. paracasei* (Table 1, Table 2).

## 3. Discussion

Experimental studies regarding the use of probiotics against helminthic infections are scarce [24]. To the best of our knowledge, this is the first study that aimed to evaluate the antiparasitic effect of *Lactobacillus* strains against *T. britovi* in a murine experimental model. The current study emphasizes the antiparasitic effect of *L. casei* and *L. paracasei* against *T. britovi* (adults) infection in mice. The mice treated with these two probiotic strains showcased an overall lower parasite count (both adults and larvae) but with statistically significant results found only in the case of adult parasites compared to the control groups. The observed differences in adult worm count could be linked to the effect of the used probiotics on the development of the larvae to the adult stage, yet not affecting the reproductive capability of the adult worms. This effect was more pronounced in female mice, which might indicate that hormonal changes that appear during their sexual cycle could influence the efficacy of some probiotics. Different mechanisms can explain this outcome—mainly, the ability of probiotics to compete with pathogens for adhesion sites, increase intestinal mucosal barrier activity, synthesize antimicrobial agents, and modulate the host’s immune system [14,15,16]. 

The mechanism behind the immunomodulation exhibited by probiotics involves interactions between these microorganisms and the gut-associated lymphoid tissue (GALT), which is an important local immune compartment. Probiotics can modulate the activity of several cells that compose GALT, such as enterocytes, dendritic cells, and T-cells, therefore increasing the protection against intestinal infections [25,26,27,28]. Furthermore, evidence regarding the role of gastrointestinal bacteria on nematode infections can be found in the correlation between microbiota composition and parasite species infections [28]. 

The results of the current study showed only a moderate inhibition capacity of *L. casei* and *L. paracasei* on *T. britovi* larvae and a stronger one against adult parasites. Considering the lack of previous research on the effects of probiotics on *T. britovi*, the interpretation of the obtained results is somewhat difficult. Therefore, several studies conducted on *T. spiralis* will be further used for comparison.

In the current study, 10^5^ CFU/mL/animal in 100 µL of Ringer’s solution was administered to each mouse. In contrast, other studies used different doses. For example, in one study, 1.9 × 10^9^ CFU/mL of viable *L. casei* was orally administered to mice seven days before *T. spiralis* infection. This latter dose induced a significantly protective response against trichinellosis [29]. 

The current experimental protocol used two strains of *Lactobacillus* (*L. casei* and *L. paracasei*) and tested the mice on days 9 and 32 p.i., whereas other similar studies included a higher number of *Lactobacillus* strains and tested the animals during other time frames. In the study by El Temsahy et al. [21], among *L. plantarum*, *L. casei*, and *L. acidophilus*, the most effective in reducing the number of *T. spiralis* larvae in male Swiss mice, on day 28 p.i., was *L. plantarum* (87%), followed by *L. casei* (75%), and finally *L. acidophilus* (61%). Dvoroznáková et al. [22] found that on day 18 p.i., the number of *T. spiralis* larvae in BALB/c male mice was under 10/mouse, as assessed by artificial digestion, in the groups treated with *L. fermentum*, *L. plantarum*, *E. faecium*, and *E. durans*. Likewise, in another study, a significant decrease in the number of muscle larvae was detected on days 25 and 32 p.i. in the groups of BALB/c male mice that received *L. fermentum* AD1-CCM7420 and *L. plantarum* 17L/1 probiotic strain, and *E. faecium* AL41 and *E. durans* ED26E/7 bacteriocin-producing strains, respectively [23]. In the case of this latter study, the production of larvae was evaluated by the reproductive capacity index (RCI). The administration of *E. faecium* CCM8558, *E. durans* ED26E/7, *L. fermentum* CCM7421, and *L. plantarum* 17L/1 strains in male BALB/c mice resulted in a significant *T. spiralis* larval reduction, with a higher efficacy seen on day 25 p.i. (13,220–23,250 larvae/mice) [23]. 

The present study also suggests that the probiotics used might influence the local immune response of the host at the intestinal level, influencing the adults’ reproductive capability. This theory is supported by other similar experimental protocols. Vargová et al. [9] showed that the administration of *E. faecium* EF55, *E. faecium* CCM7420, *E. faecium* CCM8558, *E. durans* ED26E/7, *L. fermentum* CCM7421, and *L. plantarum* 17L/1 influenced *T. spiralis* in male BALB/c mice during the intestinal and muscular phase of infection, while it may also influence the host’s macrophagic activity. Both lactobacilli stimulated the metabolic activity of macrophages during the intestinal and early muscular phase of the infection (days 5 to 25 p.i). However, in the same study, enterococci increased the O_2_- production in the intestinal phase (day 5 p.i.) and during the muscular phase (days 25 and 32 p.i.) of this parasitic infection. The detection of helper CD4, cytotoxic CD8 T lymphocytes, and B lymphocytes in the small intestine of mice treated with probiotic strains and infected with *T. spiralis* was observed in a similar experiment [30]. *Lactobacillus fermentum* CCM7421 and *L. plantarum* 17L / 1 increased the numbers of helper CD4 T cells in the epithelium and cytotoxic CD8 T cells in the lamina propria on the 7th day of administration (before parasitic infection) [30]. Moreover, an 83.3% reduction in the burden of *T. spiralis* muscle larvae was observed on day 28 p.i. in female BALB/c mice which were immune-stimulated with NC8-pSIP409-pgsA-mIL-4 (IL-4 co-expressed with pgsA anchor system of *Lactobacillus plantarum* NC8 and delivered by live *Lactobacillus plantarum* NC8) [31].

Similar studies involving the use of probiotics have been conducted on other parasitic infections. A good example is an experimental study in mice (30 Swiss female mice) infected with *Toxocara canis* (100 eggs) and inoculated with *Saccharomyces boulardii* (10^7^ CFU/g). The probiotic used promoted a reduction in the intensity of *T. canis* infection and modulated cytokine mRNA expression [32]. Another study investigated the effect of *Bifidobacterium lactis* subspecies *animalis* (3.5 × 10^10^ CFU) on the immune and intestinal function of young pigs subsequently infected with *Ascaris suum* eggs (100/animal). The probiotic treatment significantly increased mRNA expression of genes associated with enhanced protection against parasitic infection, including IL-25, RETNLB, and SOCS3, and did not interfere with the normal expulsion of L4 from the jejunum [33].

## 4. Materials and Methods

### 4.1. Animals

Male (n = 40) and female (n = 40) white CD-1 mice, six to eight weeks old, weighing 20–30 g each, were used for the present study. The decision to add female mice to the experimental protocol was taken to observe if the hormonal changes that occur during their sexual cycle would influence the efficiency of the chosen probiotics. The mice were kept in the rodent facility of the University of Agricultural Sciences and Veterinary Medicine (UASVM) Cluj-Napoca (Romania) with the approval of the Ethics Committee of the same university, through permit number 265/12.07.2021. Mice were housed in clean polypropylene-covered cages, in a well-ventilated room (24 ± 2 °C) with relative humidity (42 ± 2%) and a 12:12 h light: dark cycle. The mice were fed with standard pellet food with free access to drinking water, under strict hygiene conditions. The bedding was changed weekly. Before the experiment, the mice were given five days to adapt to the laboratory environment.

### 4.2. Probiotic Strains and Parasites

*Lactobacillus casei* ATCC 393 strain was obtained from the Department of Food Science, UASVM Cluj-Napoca, while *L. paracasei* CNCM was purchased from a local pharmaceutical company (Sofar, Bucharest, Romania).

The *T. britovi* larvae that were used during the present experiment were previously isolated from a wolf (*Canis lupus*), with the species confirmed by molecular biology (Multiplex Polymerase Chain Reaction) at UASVM Cluj-Napoca and kept alive in the Parasitology Department, Faculty of Veterinary Medicine, UASVM Cluj-Napoca (Romania), until the experiment [34].

### 4.3. Experimental Design

The experimental design and protocol were approved by the Veterinary Sanitary and Food Safety Department in Cluj-Napoca, with the permit number 277, and by the Ethics Committee of the UASVM Cluj-Napoca (Romania) and respected the national legislation (Law 43/2014) and European legislation (EU Directive 63/2010), respectively. 

Animals were randomly divided into four groups, each with two female and two male replicates at five mice/cage (n = 20) (Table 4). Probiotic strains were administered orally, in a daily dose of 10^5^ CFU/mL/animal, in 100 µL of Ringer’s solution. The mice were infected with 100 larvae/animal of *T. britovi*, on the 7^th^ day of treatment. On day 9 p.i., 10 mice (5 females and 5 males) from each group were euthanized (cervical dislocation), then their intestinal walls and intestinal content were examined. On day 32 p.i., the remaining 10 mice (5 females and 5 males) from each group were euthanized (cervical dislocation), and muscle samples were evaluated by trichinoscopy and artificial digestion.

### 4.4. Collection of Adult Parasites from the Intestinal Contents and Walls of Mice

To obtain the intestinal content, 10 mice (5 females and 5 males) were euthanized from each of the 4 groups on day 9 p.i. Afterward, the intestines were collected and cut transversally in segments of 5–10 cm. Each obtained segment was then cut longitudinally to gain access to the content and placed in a sieve in a conical container with saline (0.9% NaCl) solution. The samples were incubated at 37 °C for 4–5 h. Finally, the obtained sediment was examined with a 60× stereomicroscope (Olympus Bx61, Olympus Europe Holding, Hamburg, Germany) to count the adult parasites. The intestinal segments used in the previous step were collected and digested using the artificial digestion method as described in previously published articles and EU regulations [35], following the official protocol [34], to count the adults (females) in the intestinal mucosa.

### 4.5. Trichinoscopy and Artificial Digestion

The presence and distribution of *Trichinella* larvae in various muscle groups were determined through trichinoscopy. From each mouse, one compressor with 28 sections was examined as follows: masseter muscle −3 positions, diaphragm muscle −5 positions, forearm muscle −10 positions (5 left, 5 right), and hind leg muscles −10 positions (5 left, 5 right). The samples were examined at 4× magnification [3]. The mice carcasses (without fur, skin, and internal organs) used in the previous step were collected and digested individually with the artificial digestion method [35], following the official protocol [34], to count the larvae.

### 4.6. Statistical Analysis

Arithmetic mean and standard error of the mean were calculated for adults and larvae of *T. britovi* obtained after experimental infection in mice at 9 and 32 days p.i. by different techniques. These were computed by the gender of mice (females and males) and, in total, in the experimentally infected groups using MedCalc® Statistical Software version 20.014 [36]. Then, all data were analyzed by one-way ANOVA and Tukey–Kramer HSD test using online software. The level of significance was set at 0.05 [37].

## 5. Conclusions

The current experimental study in a murine model showed that *L. casei* ATCC 393 and *L. paracasei* CNCM are two probiotics that could potentially impact the development of the intestinal stage of *T. britovi*, although the exact mechanism behind this process needs further research. The results of the present study also indicated that *L. casei* ATCC 393 may be more efficient in reducing the number of *T. britovi* adults in female mice than *L. paracasei* CNCM.

## Figures and Tables

**Table 1:** The mean number (±SEM) of adults (9 days p.i.) and larvae (32 days p.i.) of *T. britovi* in the experimental groups.

		NC	PC	*L. casei*	*L. paracasei*	F_(2,29)_	*p*
Day 9 p.i. Adults	Intestinal content	0	41.3 ± 6.24 ^a^	15.3 ± 6.84 ^a^	40.4 ± 9.76 ^a^	3.61	0.0408
	Intestinal wall (artificial digestion)	0	24.6 ± 4.78 ^a^	6.3 ± 3.03 ^b^	11.7 ± 4.29 ^ab^	5.259	0.0118
Day 32 p.i. Larvae	Trichinoscopy	0	200.1 ± 26.98 ^a^	186.6 ± 25.76 ^a^	238.4 ± 10.32 ^a^	1.446	0.2532
	Artificial digestion	0	2967.6 ± 134.08	2452.7 ± 138.72	2895.0 ± 249.05	2.348	0.1148

SEM—standard error of mean; p.i. —post-infection; NC—negative control group, uninfected and untreated; PC—positive control group, infected with 100 larvae/animal of *T. britovi* and untreated; *L. casei*—experimental group *L. casei* ATC 393, infected with 100 larvae/animal of *T. britovi* and treated with *L. casei* 10^5^ CFU/mL in 100 µL; *L. paracasei*—experimental group *L. paracasei* CNCM, infected with 100 larvae/animal of *T. britovi* and treated with *L. paracasei* 10^5^ CFU/mL in 100 µL; *F*—statistical value (degree of freedom); *p*-value < 0.05 was considered significant. Values with no common superscript in a column within an experiment were significantly different (*p* < 0.05). ^a^-these values are not statistically significant. ^b^-These values are significantly different (*p* < 0.05) from those with superscript ^a^. ^ab^- these values are not significantly different from those with superscript ^ab^.

**Table 2:** The mean number (±SEM) of adults (9 days p.i.) and larvae (32 days p.i.) of *T. britovi* in female mice from the experimental groups.

		NC	PC	*L. casei*	*L. paracasei*	F_(2,14)_	*p*
Day 9 p.i. Adults	Intestinal content	0	42.6 ± 4.37 ^a^	7.4 ± 6.41 **^a^**	41.6 ± 16.95 **^a^**	3.467	0.0648
	Intestinal wall (artificial digestion)	0	29.0 ± 5.17 ^a^	4.8 ± 1.53 ^b^	7.4 ± 4.71 ^b^	10.331	0.0025
Day 32 p.i. Larvae	Trichinoscopy	0	223.8 ± 40.15 ^a^	212.2 ± 33.91 ^a^	221.8 ± 8.02 ^a^	0.041	0.9601
	Artificial digestion	0	2751.0 ± 203.76 ^a^	2447.4 ± 235.29 ^a^	2650.0 ± 263.63 ^a^	0.431	0.6595

*SEM*—standard error of mean; p.i. —post-infection; NC—negative control group, uninfected and untreated; PC—positive control group, infected with 100 larvae/animal of *T. britovi* and untreated; *L. casei*—experimental group *L. casei* ATC 393, infected with 100 larvae/animal of *T. britovi* and treated with *L. casei* 10^5^ CFU/mL in 100 µL; *L. paracasei*—experimental group *L. paracasei* CNCM, infected with 100 larvae/animal of *T. britovi* and treated with *L. paracasei* 10^5^ CFU/mL in 100 µL; *F*—statistical value (degree of freedom); *p*-value < 0.05 was considered significant. Values with no common superscript in a column within an experiment were significantly different (*p* < 0.05). ^a^-these values are not statistically significant. ^b^-These values are significantly different (p < 0.05) from those with superscript ^a^.

**Table 3:** The mean number (±SEM) of adults (9 days p.i.) and larvae (32 days p.i.) of *T. britovi* in male mice from the experimental groups.

		NC	PC	*L. casei*	*L. paracasei*	F_(2,14)_	*p*
Day 9 p.i. Adults	Intestinal content	0	40.0 ± 12.45 ^a^	23.2 ± 11.77 ^a^	39.2 ± 11.84 ^a^	0.621	0.5537
	Intestinal wall (artificial digestion)	0	20.2 ± 8.14 ^a^	7.8 ± 6.15 ^a^	16.0 ± 7.16 ^a^	0.768	0.4854
Day 32 p.i. Larvae	Trichinoscopy	0	176.4 ± 37.19 ^a^	161.0 ± 38.82 ^a^	255.0 ± 16.65 ^a^	2.408	0.1321
	Artificial digestion	0	3184.2 ± 126.20 ^a^	2458.0 ± 176.67 ^a^	3140.0 ± 423.79 ^a^	2.193	0.1543

SEM—standard error of mean; p.i.—postinfection; NC—negative control group, uninfected and untreated; PC—positive control group, infected with 100 larvae/100 animal of *T. britovi* and untreated; *L. casei*—experimental group *L. casei* ATC 393, infected with 100 larvae/animal of *T. britovi* and treated with *L. casei* 10^5^ CFU/mL in 100 µL; *L. paracasei*—experimental group *L. paracasei* CNCM, infected with 100 larvae/animal of *T. britovi* and treated with *L. paracasei* 10^5^ CFU/mL in 100 µL; *F*—statistical value (degree of freedom); *p*-value < 0.05 was considered significant. Values with no common superscript in a column within an experiment were significantly different (*p* < 0.05). ^a^-these values are not statistically significant.

**Table 4:** The groups in the experimental protocol, the number of animals used, the doses of probiotics used, and the number of *T. britovi* larvae used for the experimental infection.

Group	Abbreviation	No. of females	No. of males	Dose of probiotics	Experimental infection
Negative control group	NC	10	10	-	-
Positive control group	PC	10	10	-	100 larvae of *T. britovi*/animal
*L. casei* ATCC 393 experimental group	*L. casei*	10	10	10^5^ CFU/mL in 100µL	100 larvae of *T. britovi*/animal
*L. paracasei* CNCM experimental group	*L. paracasei*	10	10	10^5^ CFU/mL in 100µL	100 larvae of *T. britovi*/animal

NC—negative control group, uninfected and untreated; PC—positive control group, infected with 100 larvae/animal of *T. britovi* and untreated; CFU = colony forming units.

## Data Availability

Not applicable.
